# Modifiable factors influencing length of stay after total knee arthroplasty

**DOI:** 10.1007/s00590-022-03306-y

**Published:** 2022-06-23

**Authors:** Martin Missmann, Jean-Pascal Grenier, Christoph Raas

**Affiliations:** 1 Austrian Workers’ Compensation Board (AUVA), Ingenieur-Etzel-Str. 17, 6020 Innsbruck, Austria; 2 Department of Orthopaedics and Traumatology, Medical University of Innsbruck, Anichstr. 35, 6020 Innsbruck, Austria; 3 Department of Trauma Surgery, Privatklinik Hochrum, Laerchenstr. 41, 6063 Rum, Austria

**Keywords:** Rapid recovery surgery, Total knee arthroplasty, Length of stay, Risk factors

## Abstract

**Purpose:**

This cohort study aims to investigate the current Rapid-Recovery-(RR)-pathway at an orthopaedic surgery hospital centre and to identify preoperative, intraoperative, and postoperative factors that are significantly associated with prolonged hospital Length of Stay (LOS) after total knee arthroplasty (TKA).

**Method:**

A total of 194 patients undergoing primary TKA were included in this retrospective study. Sociodemographic data documented were age, gender, body mass index, living situation, and the clinical diagnosis. Factors affecting patient constitution and laboratory data for serum level of Hb and CRP were assessed preoperatively and postoperatively. In addition, we collected patients’ data for attendance of patient education, planned discharge to rehabilitation facilities, and levels of postoperative pain.

**Results:**

In univariate group comparisons, prolonged LOS was significantly associated with increased age, elevated C-reactive-Protein-level, and decreased haemoglobin level. Patients experiencing prolonged LOS also showed significant association with higher prevalence of comorbidities, female gender, living as widow, preoperative anticoagulation, requirement of blood transfusion, and planned discharge to rehabilitation facilities. However, after multivariate logistic regression, only planned discharge to rehabilitation facility, non-attendance of preoperative patient education, female gender, and increased pain levels were identified as significant predictors for prolonged LOS.

**Conclusion:**

Efficient pain therapy and thorough patient education have a positive effect on treatment outcome after TKA in a RR-setting.

## Introduction

Total knee arthroplasty (TKA) is a safe and effective therapy for end-stage osteoarthritis of the knee, offering long-term pain relief, improved joint function and better quality of life for patients [1]. Recent trends toward short-stay protocols for patients undergoing TKA concern the expenses of health care, but also improvements in surgical technique and perioperative care [2]. The scientific work of Danish surgeon Henrik Kehlet made a major contribution to the enhanced recovery after surgery concept, occasionally referred to as “fast track” or “rapid recovery” (RR) surgery [3]. Reducing length of stay (LOS) after TKA can be achieved by incorporating a multimodal interdisciplinary and patient-centred treatment [4]. Patients undergoing TKA under RR-conditions show reduced intake of analgesic drugs, fewer adverse effects, and report higher contentment [5].

In 2020, Wainwright et al. summarized recommendations of the perioperative care of patients undergoing THA and TKA in a multidisciplinary consensus review [6]. They include, amongst other recommendations, preoperative education for patients, the use of local anaesthetics for infiltration analgesia within a multimodal opioid-sparing analgesic regimen, and early mobilization. This study aims to investigate previous assumptions of RR models and to identify preoperative, intraoperative, and postoperative factors that are significantly associated with prolonged LOS.

## Method

**Fig. 1:**
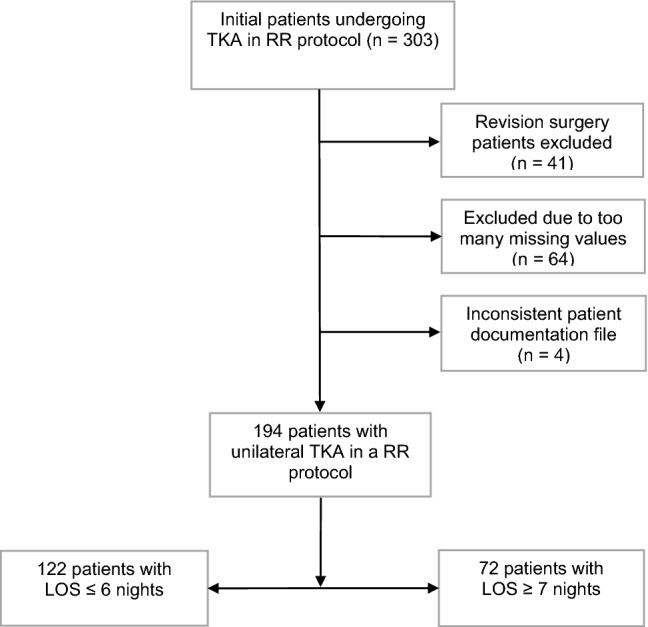
Flow chart of participants

Initially, we screened 303 subjects that underwent TKA between July 2015 and July 2019 for this retrospective cohort study. All surgeries were performed in a RR-setting. In order to obtain a homogenous sample, revision surgeries were excluded (*n* = 41) as well as cases with too many missing values (*n* = 64, then not listed in the National Joint Registry due to incomplete WOMAC data) and inconsistent data in computer patient databases (*n* = 4) (Fig. 1). Due to many missing values of CRP, pain (NPRS) and haemoglobin (Hb), categorization of these variables was performed, in order to prevent case dropouts in advanced statistical analysis.

A total of 194 patients were included in this study (55.67% female, 44.33% male). Mean age for all patients was 69.1 years, ranging from 36 to 87 years (Table 1). Mean body mass index (BMI) was 27.98 kg/m^2^, ranging from 18.4 (underweight) to 44.1 (obese class III). Patients were invited to attend a preoperative patient education program (1.5 h). In addition, we ascertained requirement for assistance at home or transferring to a rehabilitation clinic. Arterial hypertension was the comorbidity with the highest prevalence (52.6%). Preoperative anticoagulation was a present condition in 59 (30.4%) of patients, 28 (14.4%) were classified as preoperatively anaemic, and 13 (6.7%) were admitted with elevated CRP-level prior to surgery. One hundred and two patients (52.6%) attended preoperative patient education.

**Table 1:** Patients characteristics, comorbidities, and group differences separated by LOS ≤ 6 and ≥ 7

Variable	LOS ≤ 6	LOS ≥ 7	Total	Group differences
	*n* = 122 (%)	*n* = 72 (%)	*n* = 194 (%)	*p* value (95% CI)
Age (years)	67.92 ± 9.70	71.14 ± 10.21	69.11 ± 9.98	.030* (− 6.12, − .32)
Gender				.039*
Male	61 (50.0)	25 (34.7)	86 (44.3)	
Female	61 (50.0)	47 (65.3)	108 (55.7)	
BMI (kg/m^2^)	27.97 ± 4.06	27.99 ± 5.19	27.98 ± 4.50	.970 (− 1.44, 1.39)
Living situation				.090
Married	67 (54.9)	37 (51.4)	104 (53.6)	
Solitary	32 (26.2)	14 (19.4)	46 (23.7)	
Divorced	15 (12.3)	7 (9.7)	22 (11.3)	
Widowed	7 (5.7)	13 (18.1)	20 (10.3)	
Diagnosis				.725
Primary Osteoarthritis	97 (79.5)	55 (76.4)	152 (78.4)	
Posttraumatic	4 (3.3)	4 (5.5)	8 (4.1)	
Secondary posttraumatic	21 (17.2)	13 (18.1)	34 (17.5)	
Comorbidities				
Diabetes mellitus	16 (13.1)	15 (20.8)	31 (16.0)	.156
Cancer disease	17 (13.9)	9 (12.5)	26 (13.4)	.831
Apoplexy	7 (5.7)	7 (9.7)	14 (7.2)	.300
Hypertension	58 (47.6)	43 (59.7)	101 (52.1)	.101
ASA classification				
ASA I and II	105 (88.2)	52 (74.3)	157 (80.9)	.014*
ASA III	14 (11.8)	18 (25.7)	32 (16.5)	.014*
ACCI	3.95 ± 2.14	5.07 ± 2.31	4.36 ± 2.26	< .001* (− 1.78, − .46)

Values are total count with percentages in parentheses; asterisks indicating significance

BMI, Body-Mass-Index; ASA, American Society of Anaesthesiologists; ACCI, Age-adjusted Charlson-Comorbidity Index

Tranexamic acid (15–20 mg/kg body weight) and dexamethasone (16 mg intravenous) were administered to the patient 30 min prior to surgery, while benzodiazepines were not delivered at any time. None of the patients suffered from tuberculosis or an active fungal or viral infection. There was also no known subarachnoid haemorrhage or increased intravascular coagulation tendency in the anamnesis. All patients received high volume local infiltration analgesia (LIA) (113–188 mg ropivacaine or 150–250 mg bupivacaine and epinephrine) during surgery. Postoperatively, they were administered paracetamol, 2 mg oral hydromorphone on operation day (OPD), and 2 × 2 mg oral hydromorphone on postoperative day 1 (POP1). Patients with severe pain received additional opioids (1.3 mg hydromorphone intravenous every 4–6 h) on OPD and POP1.

First mobilization with was performed on the day of surgery (OPD) if possible. No surgical drains were used postoperatively. Patients were cleared for return to the ward as soon as soon as the legs could be moved and the operated leg could be lifted out of bed. Physiotherapy sessions were scheduled once daily with focus on active measures and mobilization. Nursery care instructed patients how to self-administer low-molecular weight heparin (enoxaparin 40 mg), which was prescribed for four weeks after surgery.

We encouraged patients to wear their private wardrobe and take their meals in the commensal room and not in bed. For pain management, a standardized protocol of oral postoperative medication was followed, whereas individual adjustments were necessary at times. Target discharge for the patients was defined prior to or during admission. To be discharged, patients had to be capable of getting out of bed, going to the bathroom, climbing the stairs, and walking independently through the hallway twice. We discharged patients on postoperative day 4 or 5 if the clinical and functional discharge criteria were met.

### Statistical analysis

Sociodemographic data documented for this trial were age, gender, body mass index (BMI), living situation, and the clinical diagnosis. In addition, preoperative factors affecting patient constitution were recorded such as smoking, alcohol consumption, anticoagulation, attendance of patient education, and prevalence of comorbidities. Various factors of the surgical procedure were noted as well. Serum level of Hb and CRP were assessed preoperatively and postoperatively. (Table 2). Planned discharge to rehabilitation facilities, time between surgery and initial mobilization, 30-day readmissions, and postoperative pain-management were the postoperative variables considered in this trial. LOS greater than the median was defined as prolonged LOS. Postoperative pain was categorized into missing values, acceptable pain (NPRS = 0–3), and increased pain (NPRS ≥ 4). To include comorbidities in the analysis, the American Society of Anesthesiologists (ASA) physical status classification system was obtained from surgery protocols [7], where every patient was categorized into ASA I, II, and III. In addition, we assessed comorbidities and calculated the Age-adjusted-Charlson-Comorbidity-Index (ACCI) [8].

**Table 2:** Inflammation markers and Hb-levels and group differences separated by LOS ≤ 6 and ≥ 7

Variable	LOS ≤ 6	LOS ≥ 7	Total	Group difference
	*n* = 122 (%)	*n* = 72 (%)	*n* = 194 (%)	*p* value (95% CI)
CRP preoperatively (mg/dl)	.41 ± 0.71	.44 ± 0.79	.42 ± .74	.765
Normal CRP (0.01 – 1.00)	114 (93.4)	67 (93.1)	181 (93.3)	
Elevated CRP (> 1.01)	8 (6.6)	5 (6.9)	13 (6.7)	
Hb preoperatively (g/dl)	14.28 ± 1.24	13.87 ± 1.26	14.13 ± 12.56	.028*
Hb missing	2 (1.6)	1 (1.4)	3 (1.5)	
Hb normal (≥ 13.0)	104 (85.2)	59 (81.9)	163 (84.0)	
Anaemia (< 12.9)	16 (13.1)	12 (16.7)	28 (14.4)	
Hb POPD1 (g/dl)	11.14 ± 1.42	10.53 ± 1.32	10.91 ± 14.11	.006*
Hb missing	17 (13.9)	8 (11.1)	25 (12.9)	
Mild or no anaemia (≥ 10.0)	84 (68.9)	43 (59.7)	127 (65.5)	
Severe postoperative anaemia (< 9.9)	21 (17.2)	21 (29.2)	42 (21.6)	
Hb POPD3 (g/dl)	10.25 ± 1.59	9.63 ± 1.30	10.01 ± 15.16	.016*
Hb missing	32 (26.2)	17 (23.6)	49 (25.3)	
Mild or no anaemia (≥ 10.0)	47 (38.5)	22 (30.6)	69 (35.6)	
Severe postoperative anaemia (< 9.9)	43 (35.2)	33 (45.8)	76 (39.2)	
CRP POPD3 (mg/dl)	8.25 ± 4.97	13.15 ± 8.32	10.05 ± 6.79	< .001*
CRP missing	34 (27.9)	21 (29.2)	55 (28.4)	
CRP elevated (1.00 – 9.9)	36 (29.5)	14 (19.4)	50 (25.8)	
CRP highly elevated (10.00 – 19.9)	48 (39.3)	27 (37.5)	75 (38.7)	
CRP very highly elevated (> 20.00)	4 (3.3)	10 (13.9)	14 (7.2)	
CRP POPD5 (mg/dl) *n* = 62	5.42 ± 3.59	7.62 ± 4.84	6.43 ± 4.33	.008*
Hba1c (%)	5.63 ± .95	5.86 ± .80	5.71 ± .90	.107

CRP, C-reactive Protein; Hb, Haemoglobin; POPD1, postoperative day 1; POPD3, postoperative day 3; POPD5, postoperative day 5. Continuous variables are reported as mean ± standard deviation (SD) whereas categorical variables are reported as counts and percentages in parentheses; asterisks indicating significance

Upon dichotomization of patients, sample Student’s t test was computed for continuous data to determine the relationship of continuous variables and prolonged LOS. We assessed possible relations between categorical data and prolonged LOS using the Chi-square test or Fisher’s exact test (if an expected or observed value is ≤ 5). Following investigation of univariate analysis of the independent variables and their potential relationship with LOS, a multivariate logistic regression analysis was performed to identify significant preoperative predictors for prolonged LOS. We then calculated a second multivariate logistic regression on postoperative day 3 (POPD3), in order to integrate intra- and postoperative predictors in the predictive model. Statistical analysis was performed using SPSS version 26 (IBM, New York, US), Excel 2016 (Microsoft, Redmond, WA), and Word 2016 (Microsoft, Redmond, WA).

## Results

**Fig. 2:**
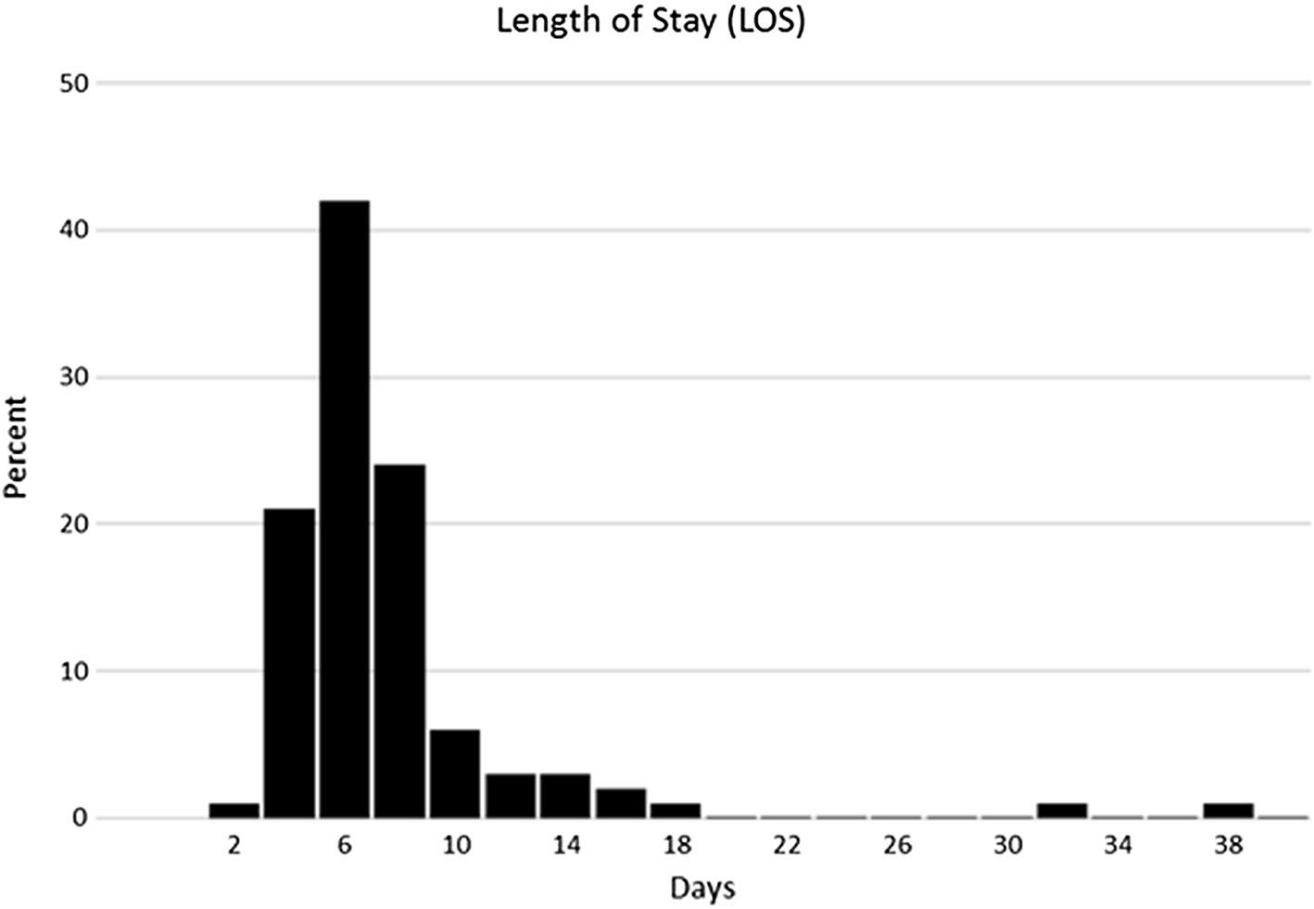
Length of stay (days)

Median LOS for patients in this study was 6.00 nights, ranging from 2 to 38 nights (Fig. 2), whereas mean LOS was 6.54 nights. Patients with ASA grade III made up 16.5% of our sample as opposed to 80.9% of patients that were classified as ASA I or II. There were 122 patients (62.88%) with LOS ≤ 6 nights and 72 patients (37.12%) with LOS ≥ 7 nights. Most patients (*n* = 177; 91.2%) could be discharged within 9 nights. Only 17 patients (8.8%) stayed in hospital for 10 nights or longer.

The majority of patients (55.2%) preferred general anaesthesia. A tourniquet was employed on 118 (60.8%) of our patients (Table 3). Most patients were mobilized on the day of surgery (66.5%). Fifteen (7.3%) of patients required intra- or postoperative blood transfusions. Mean NPRS for all patients during their LOS was 1.53 (± .55). Planned discharge to a rehabilitation facility was scheduled for 48 (24.7%) patients in this sample. Eight (4.1%) patients were readmitted 30 days after discharge.

**Table 3:** LOS and factors during surgery: univariate analysis of categorical variables

Variable	*χ*^2^ (df)	*p*	OR for LOS ≥ 7
Gender	4.282 (1)	.039*	1.88 for female gender
Living situation	7.923 (3)	.048*	3.95 for widows over divorced 3.38 for widows over married 4.32 for widows over solitary
Diagnosis	.644 (2)	.725	n.s
Season of surgery	5.325 (3)	.150	n.s
Weekday of surgery	.798 (3)	.850	n.s
PrOP anticoagulation	4.160 (1)	.041*	1.91 for anticoagulated patients
Diabetes mellitus	2.009 (1)	.156	n.s
Apoplexy	1.074 (1)	.300	n.s
Cancer disease	.080 (1)	.831	n.s
Hypertension	2.692 (1)	.101	n.s
Blood transfusion	12.811 (1)	< .001**	7.93 for patients who received blood transfusion
Type of anaesthesia	.262 (1)	.609	n.s
Planned rehab	7.947 (1)	.005*	2.57 for patients with planned rehab
PrOP Patient-education	2.920 (1)	.087	n.s
Tourniquet	.135 (1)	.713	n.s
Smoking	3.606 (1)	.058	n.s
Alcohol	2.725 (1)	.099	n.s
First mobilisation	.076 (1)	.783	n.s
ASA III	6.098 (1)	.014*	2.97 for patients with ASA III over patients with ASA I or II
Cement used	2.393 (2)	.302	n.s
Right or left leg	.088 (1)	.766	n.s

### Factors associated with prolonged LOS

Increased age was associated with prolonged LOS as patients with prolonged LOS were older (71.14 ± 10.21) than patients with normal LOS (67.92 ± 9.70). Females tended to have greater LOS than males. The ACCI was significantly higher in the prolonged LOS cohort. As increased ACCI-score was associated with prolonged LOS, ASA III classification increased the odds for prolonged LOS (OR = 2.97). BMI was not associated with prolonged LOS in this study. Widowed patients had an increased risk of prolonged LOS compared to solitary patients (OR = 4.32), or divorced (OR = 3.95), or married patients (OR = 3.38) (Table 1).

In addition, laboratory data and pain showed significant associations with prolonged LOS. Anticoagulated patients were two times (OR = 1.91) more likely to experience prolonged LOS, while patients that required blood transfusions were eight times (OR = 7.93) more likely to experience prolonged LOS. Patients with preoperative anaemia, decreased Hb on POPD1, and on POPD3 showed a significant relationship with prolonged LOS. Higher CRP-level on POPD5 resulted in increased risk for prolonged LOS (Table 2). Increased pain on POPD1 and POPD3 was associated with prolonged LOS. A prolonged LOS became 2.57 times more likely when a discharge to a rehabilitation facility was planned.

### Predicting prolonged LOS

Interestingly, several factors, which showed significant association with prolonged LOS in univariate analysis, were no longer significant in multivariate analysis. The first model was calculated including gender, age, living situation, ASA, planned discharge to rehabilitation facility, preoperative patient education, ACCI, preoperative Hb and CRP values. This resulted in a significant model (*p* < .001) with a *χ*^2^ = 48.70 and an ability to correctly classify the patients in 74.2%. Three preoperative factors were identified as significant predictors for prolonged LOS:

- planned discharge to a rehabilitation facility after surgery (*p* = .013);
- not attending preoperative patient education (*p* = .034);
- female gender (*p* = .047).

**Fig. 3:**
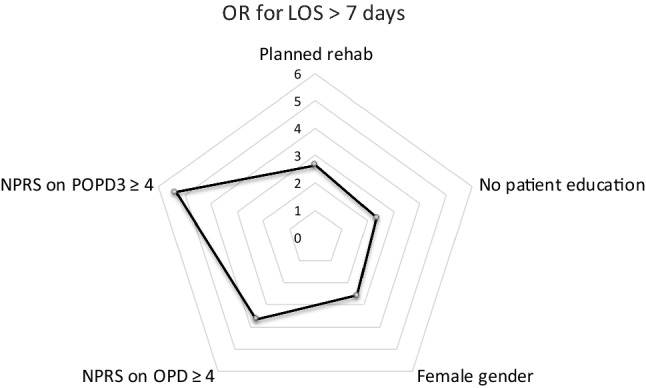
Multivariate analysis of prolonged LOS

The building method of the second model was identical to the first one, but additionally including pain measures (NPRS) on OPD, POPD1 and POPD3. This resulted in another significant model (*p* < .001) with *χ*^2^ = 45.57 and an ability to correctly classify the patients in 77.8%, thereby only providing a slightly superior predictive model compared to the preoperative (74.2%) one (Fig. 3). Patients that experienced increased pain on the day of surgery were more than three times as likely to experience prolonged LOS. Patients with increased pain on POPD3 were even five times as likely to experience prolonged LOS. Five factors were identified as significant predictors for prolonged LOS in the postoperative multivariate analysis:

- female gender (*p* = .036);
- planned discharge to a rehabilitation facility after surgery (*p* = .020);
- not attending preoperative patient education (*p* = .050);
- reported increased pain (NPRS of ≥ 4) on OPD (*p* = .022);
- reported increased pain on POPD3 (*p* = .017).

## Discussion

The most vital finding was the identification of several factors as significant predictors for prolonged LOS (≥ 7 nights), such as female gender, planned discharge to a rehabilitation facility, non-attendance of preoperative patient education, increased pain on OPD and on POPD3. Compared to the results of other studies, the median of the LOS after TKA for all patients included in this study (6.00 nights) can be considered high and reflects regional health care conditions in Central Europe [9]. We could not detect a significant association between LOS and the following parameters, such as season of surgery, operative time, HbA1c-level preoperatively, presence of selected comorbidities (diabetes mellitus, apoplexy, hypertension, and cancer disease), and usage of tourniquet, which can lead to adverse effects, including impairment of muscle strength and vascular and nerve-related alteration. Therefore, in our protocols, a tourniquet was used only in cases of significant tissue bleeding or trabecular haemorrhage. As mentioned above, LOS was not significantly influenced by this measure, which is consistent with the results of a systematic review on this topic by McCarthy et al. [10].

### Patient demographics associated with prolonged LOS

Comorbidities, increasing age, and female gender are considered as non-modifiable patient factors and were significantly associated with prolonged LOS in our sample. This pattern of results is in line with the previous literature, including a recently published systematic review, showing an association with prolonged LOS after TKA [11]. While increasing age is a significant predictor of prolonged LOS in other studies, this is not the case in our patient population and could be explained by the small number of participants aged ≥ 80 years with only 25 patients (12.8%) across both LOS cohorts. As for comorbidities, ASA and ACCI showed significant association with prolonged LOS in univariate analyses. However, after they were included in the multivariate analysis, they were no longer significant, which is in contrast to a recently published observational study on factors associated with LOS in TKA patients [12].

### 30-day readmission rate

30-day readmission rate (total 4.12%, 8 cases) was significantly higher in the prolonged LOS cohort (6.9%, 5 cases) than the normal LOS cohort (2.5%, 3 cases). There exists a body of research investigating the relationship of reduced LOS and readmission rates [13]. Compared to this, the readmission rates in our patient population appear to be rather low. To a certain extent, even a further reduction in LOS after primary TKA in a RR-setting appears to be safe and does not increase short-term readmission rates [14].

### Preoperative patient education

Patients who did not attend preoperative patient education (*n* = 58) were 2.36 as likely to experience prolonged LOS compared to those who did (*n* = 106). While non-significant in univariate analysis (*p* = .087), multivariate analysis identified preoperative patient education as a significant predictor for prolonged LOS (*p* = .050). This is in contrast to the findings of other studies, in which preoperative patient education did not offer any additional benefit with regard to the functional outcome [15, 16]. On the other hand, our results and findings from other research groups indicate that preoperative patient education is significantly associated with reduced LOS and improved outcome after TKA [17]. Patient education prior to TKA is unlikely to cause harm and is potentially anxiety relieving. In their recently published systematic review, Wainwright and colleagues put forth a strong recommendation for including preoperative patient education in RR concepts for TKA [6].

### Pain management

Effective postoperative pain management during the first 24 h after surgery is essential, as reduced pain-scores over this period have shown to improve short- and medium-term functional outcome. Increased pain-levels (NPRS ≥ 4) on OPD and POPD3 were identified as significantly predicting prolonged LOS after TKA. In a systematic review by Li and colleagues, multimodal postoperative analgesic regimens were confirmed as best practice for patients after TKA [18]. Patients in our study were postoperatively administered paracetamol and hydromorphone, in part as patient-controlled analgesia with the acceptance of the well-known side effects of short-term opioid therapy. However, pre-emptive analgesia was not considered and could be an important additional component of pain therapy in the future.

### Psychological factors

Besides the living situation, no psychosocial factors were obtained from our patients. Considering that TKA patients have been reported to show signs of mental distress prior to and after surgery, the lack of psychosocial data is worrisome. Poor preoperative psychological health was identified as a risk factor for prolonged LOS after TKA in a recently published systematic review [19]. Similarly, Napier et al. identified social reasons as the most common cause for prolonged LOS in TKA patients [20].

### Living situation

In our sample, patients living alone have prolonged LOS after TKA, which is in accordance with a study by Husted et al., including more than 700 consecutive patients [21]. Widowed patients were approximately four times as likely to experience prolonged LOS compared to married, divorced, and solitary patients. The most compelling explanation for this finding could be progressing age, as widowed patients generally tend to be older (76.2 years ± 6.32) than sample age average (69.11 years), and higher prevalence of comorbidities in patients with increased age. Tukey post hoc analysis revealed widowed patients to have increased ACCI-scores compared to solitary patients and compared to divorced patients. Consequently, the increased OR in widowed patients for prolonged LOS after TKA can be explained by increasing age and higher prevalence of comorbidities in this population group.

## Limitations of this study

Potentially limiting is the amount of missing values in certain variables, which ultimately led to loss of information because categorizations of several independent variables were conducted in order to prevent high dropout-rates for multivariate analysis. In addition, no information about physical fitness other than information about comorbidities and BMI was available, for instance data on quadriceps strength and function. Another limitation is the lack of inclusion of several psychological and social factors. Besides the living situation, no other psychosocial factors were obtained from the patients. Further research is recommended using valid and viable questionnaires to identify patients in distress. Implementation of such tools should be sought after by clinicians to extend the prevailing reductionist biomechanical explanations for systemic pathologies such as osteoarthritis [22].

## Conclusions

The present study has identified female gender, planned discharge to rehabilitation facilities, non-attendance of preoperative patient education, and increased pain levels (NPRS ≥ 4) on the day of surgery and on postoperative day 3 as significant predictors for prolonged LOS after TKA in a RR-setting.
